# Transmission dynamics of SARS-CoV-2 in British Columbia’s largest school district during the second half of the 2020–2021 school year

**DOI:** 10.17269/s41997-022-00659-z

**Published:** 2022-07-14

**Authors:** Laurence Campeau, Frances Thistlethwaite, Jiayun Angela Yao, Amy J. Hobbs, Armin Shahriari, Rohit Vijh, Carmen H. Ng, Christina Fung, Shannon Russel, James Zlosnik, Natalie Prystajecky, Ariella Zbar

**Affiliations:** 1 Public Health Agency of Canada, Ottawa, Ontario Canada; 2 Office of the Medical Health Officer, Fraser Health, Surrey, British Columbia Canada; 3 British Columbia Observatory for Population and Public Health, BC Surrey, Canada; 4 School of Population and Public Health, University of British Columbia, Vancouver, British Columbia Canada; 5 British Columbia Centre for Disease Control Public Health Laboratory, Vancouver, British Columbia Canada; 6 Department of Pathology and Laboratory Medicine, University of British Columbia, Vancouver, British Columbia Canada

**Keywords:** Canada, COVID-19, Schools, Risk factors, Canada, COVID-19, écoles, facteurs de risque

## Abstract

**Objectives:**

To determine the extent and characteristics of in-school transmission of SARS-CoV-2 and determine risk factors for in-school acquisition of COVID-19 in one of Canada’s largest school districts.

**Methods:**

We conducted a retrospective chart review of all reportable cases of COVID-19 who attended a kindergarten–Grade 12 (K-12) school within the study area between January and June of the 2020–2021 school year. The acquisition source was inferred based on epidemiological data and, when available, whole genome sequencing results. Mixed effects logistic regression was performed to identify risk factors independently associated with in-school acquisition of COVID-19.

**Results:**

Overall, 2877 cases of COVID-19 among staff and students were included in the analysis; of those, 9.1% had evidence of in-school acquisition. The median cluster size was two cases (interquartile range: 1). Risk factors for in-school acquisition included being male (adjusted odds ratio [aOR]: 1.59, 95% confidence interval [CI]: 1.17–2.17), being a staff member (aOR: 2.62, 95% CI: 1.64–4.21) and attending or working in an independent school (aOR: 2.28, 95% CI: 1.13–4.62).

**Conclusion:**

In-school acquisition of COVID-19 was uncommon during the study period. Risk factors were identified in order to support the implementation of mitigation strategies that can reduce transmission further.

## Résumé

**Objectifs:**

Déterminer l’étendue et les caractéristiques de la transmission de la SRAS-CoV-2 en milieu scolaire, et déterminer les facteurs de risque de l’acquisition de la COVID-19 dans l’un des plus larges arrondissements scolaires du Canada.

**Méthodes:**

Nous avons mené un examen rétrospectif des dossiers de tous les cas signalés de COVID-19 ayant fréquenté une école de niveau élémentaire, primaire ou secondaire dans la zone à l’étude entre janvier et juin de l’année scolaire 2020-2021. La source d’acquisition était inférée sur la base des données épidémiologiques et, lorsque disponibles, les résultats de séquençage du génome entier. Nous avons eu recours à des régressions logistiques multiniveaux pour identifier les facteurs indépendamment associés avec l’acquisition de la COVID-19 en milieu scolaire.

**Résultats:**

Au total, 2 877 cas de COVID-19 parmi les employés et les élèves ont été inclus dans l’analyse; de ceux-ci, 9,1 % avaient acquis l’infection en milieu scolaire. La grosseur médiane des agrégats était de deux cas (écart interquartile : 1). Les risques facteurs de l’acquisition en milieu scolaire incluaient le fait d’être de sexe masculin (rapport de cotes ajusté [RCa] : 1,59, intervalle de confiance [IC] de 95% : 1,17-2,17), être un membre du personnel (RCa : 2,62, IC de 95% : 1,64-4,21) et fréquenter ou travailler dans une école indépendante (RCa : 2,28, IC de 95% : 1,13-4,62).

**Conclusion:**

Nos résultats suggèrent que l’acquisition de la COVID-19 en milieu scolaire était peu commune pendant la période d’étude. Des facteurs de risque ont été identifiés afin de supporter l’implémentation de mesures de contrôle pouvant réduire davantage la transmission.

## Introduction

There is emerging evidence that disruptions to in-person primary and secondary school instruction during the coronavirus disease (COVID-19) pandemic have brought about high social and economic costs to communities. Children who experienced school closures not only suffered from a loss of learning (Engzell et al., 2021), but also had their physical and mental health impacted (Chaabane et al., 2021). This is especially true for children from disadvantaged backgrounds, who are more likely to experience pandemic-related disruptions in intersection with other adverse circumstances, such as home instability and food insecurity (Hawrilenko et al., 2021). The closures are also believed to have important consequences on the economy; the impact of school closures for a third of the year is predicted to amount to a loss of $1,272 billion and $14,197 billion for Canada and the United States, respectively, due to a lower achieving labour force (Hanushek & Woessmann, 2020).

The impact of school closures must be balanced against the risk that educational institutions present in terms of transmission of severe acute respiratory syndrome coronavirus 2 (SARS-CoV-2). While various studies have shown that transmission in this setting is generally a reflection of the level of transmission in surrounding communities (Goldhaber et al., 2021; Ismail et al., 2021; Russell et al., 2020), a number of outbreaks in the school setting have been identified at different time points during the pandemic (Kampe et al., 2020; Lam-Hine et al., 2021; Stein-Zamir et al., 2020).

In the Canadian context, evidence remains sparse as to the degree to which transmission of COVID-19 occurs in kindergarten to Grade 12 (K-12) schools. To our knowledge, only one peer-reviewed observational study has been published. The study, which took place in Vancouver, British Columbia, showed that only 8.1% of cases of COVID-19 among school-based individuals had been acquired in the school setting in the 3 months following the re-opening of schools in September 2020 (Bark et al., 2021). In the present study, our objective was to determine the extent of in-school transmission of SARS-CoV-2 within the largest school district of British Columbia, Canada, and to investigate risk factors associated with in-school acquisition of COVID-19.

## Methods

### Study design and setting

We conducted a retrospective chart review of all COVID-19 cases who either worked at or attended a K-12 school within the geographic boundaries of Surrey school district during the second half of the 2020–2021 school year. This school district is located in the region covered by Fraser Health, one of the five regional health authorities in British Columbia, and constitutes the largest school district in the province in terms of school population (Government of British Columbia, n.d.). It serves more than 74,000 students from 135 public K-12 schools distributed across the cities of Surrey and White Rock, and the rural area of Barnston Island (Surrey School District, n.d.). An additional 34 independent schools (not part of the public school system) and one public school operated by the province’s French-language school board are located within its geographic boundaries and have been included in the present study (British Columbia Ministry of Education, email communication, January 2021).

During the study period, general public health measures included restrictions on personal gatherings, a ban on indoor dining and limits on intra-provincial travel. Some of these measures were gradually loosened at the end of May 2021 as part of a provincial restart plan. While short-term school closures episodically took place in response to school-based transmission of COVID-19 or staff shortages, the majority of schools remained open for the duration of the study period. On March 27, 2021, the use of non-medical masks became required within learning groups for staff and students in Grade 4 and above. For younger children, the use of non-medical masks was not required, but an update to the provincial guidelines published on March 30, 2021, encouraged their use. All staff and students who were found to be in close contact to a case of COVID-19 at school were instructed to get a COVID-19 test, whether they experienced symptoms or not. Other measures included in the provincial COVID-19 health and safety guidelines for K-12 settings are summarized in Appendix 1.

Staff members working in schools in the study area were the first to be part of a mass vaccination campaign of school-based staff that took place in late March 2021. Approximately 85% of staff in the area had received a first dose of a vaccine by April 7, 2021 (Fraser Health, unpublished data, 2021). Students aged 12 and older across the province subsequently became eligible for vaccination on May 19, 2021.

### Case and cluster definitions

Individuals were considered for inclusion in the study if they met one of the reportable case definitions for COVID-19 established by the British Columbia Centre for Disease Control (BCCDC). This includes confirmed, probable-laboratory and probable-epi-linked cases (BCCDC, n.d.-a). Cases were included if (1) they attended or worked at a public or independent K-12 school located within the geographic boundaries of Surrey school district, and (2) they attended school in person during their acquisition or infectious period during the second half of the 2020–2021 school year, which took place between January 4 and June 25, 2021. Staff members in all capacities were included (e.g. teacher, administrative assistant, principal, etc.). A cluster of cases was defined as two or more cases of COVID-19 who attended or worked at the same school and were found to have transmitted to each other in the school setting. Within each cluster, cases were classified as either primary (the first person to bring the virus into a group of people) or secondary (the cases arising from the primary case). Outbreaks were declared by the Medical Health Officer when there was sustained, uncontrolled and widespread transmission of COVID-19 within a school, and extraordinary public health measures were necessary to stop further transmission.

### Whole genome sequencing

Whole genome sequencing (WGS) was performed at the BCCDC Public Health Laboratory (PHL) (Vancouver, BC, Canada), which serves as the reference laboratory for the province. In brief, SARS-CoV-2-positive specimens were sequenced using a tiled 1200-bp amplicon scheme and sequenced on either an Illumina MiSeq or NextSeq. Of the 2877 cases included in the study, a subsample of 2399 SARS-CoV-2-positive specimens underwent WGS analysis, 78.2% (*n*=1876) of which were successfully sequenced. This resulted in 64.6% (*n*=1654) of students and 66.6% (*n*=221) of staff having valid WGS results. Phylogenetic trees were constructed using kovid-trees-nf (Goncalves da Silva & Seemann, 2021) modified into a snakemake workflow, followed by clade assignment using the Tree Cluster algorithm using a maximum clade size of 6 mutations separating samples with the same Clade number (Balaban et al., 2019). Cases in the same clade were considered related to each other if this was consistent with the epidemiological data.

### Detection of variants of concern (VoC)

Genetic characterization was also performed at the BCCDC PHL and was based on a combined VoC testing strategy that used both WGS and/or targeted VOC single-nucleotide polymorphism (SNP) quantitative polymerase chain reaction (qPCR). Cases were classified as having a VoC (Alpha, Beta, Gamma or Delta) based on the SNP profile (when tested by qPCR) and/or lineage determination from whole genome sequencing. This approach has been detailed elsewhere (Hogan et al., 2021).

### Classification of source of acquisition

We used case-level epidemiological data, including exposure information, contact tracing data, and symptom onset dates (specimen collection dates for asymptomatic cases), to determine whether COVID-19 infection was acquired in school settings. Cases were classified as having evidence of in-school acquisition if they (1) attended school during their acquisition period, (2) did not have any other known possible acquisition source (e.g. household or community), and (3) were in close contact with an infectious case of COVID-19 in the school setting, or their acquisition period overlapped with the infectious period of another case in the same classroom or administrative area. More information on the criteria used to determine the source of acquisition can be found in Appendix 2.

WGS data, when available, were used to confirm or rule out transmission links between cases. Due to the wide genetic diversity of SARS-CoV-2 lineages circulating in the region during the study period, two cases within a clade who did not report being in contact in other settings were considered as having transmitted within the school if this was consistent with the epidemiological data. Cases that were initially classified as having transmitted to each other in the school setting based on epidemiological review but did not share a clade were reclassified as having an unknown source of acquisition.

### Data collection

Data on COVID-19 cases were collected using the Primary Access Regional Information System (PARIS), the clinical information system used by Fraser Health for case and contact management of COVID-19. As part of routine public health follow-up, each newly diagnosed case is contacted to collect information on acquisition source, symptoms, contacts, school attendance dates and all potential transmission events during the case’s infectious period. Personal health numbers (PHN) were used to link each case with their vaccination status and whole genome sequencing results. A standardized data collection template was developed and used by a team of epidemiologists and data analysts to categorize the cases’ likely source of acquisition and onward transmission events. To ensure a high degree of consistency between reviewers, a subset of cases were independently reviewed by two team members, and discrepancies between the decisions were discussed as a team to achieve full agreement. Additionally, regular meetings to discuss complex cases with the Medical Health Officer were held.

### Statistical analyses

For descriptive statistics, continuous variables are presented as means (standard deviation [SD]) or medians (range) and categorical variables are presented as numbers and percentages. Pearson’s chi-squared (*χ*^2^) tests were used to identify statistically significant differences between students and staff. *P*-values below 0.05 were considered statistically significant.

To calculate the incidence of COVID-19, the population denominator for each Community Health Service Area (CHSA), which represents the most granular health administrative boundary defined by the British Columbia Ministry of Health, was extracted from BC Stats Population Estimates (BC Stats 2021). Data on the 2020–2021 student enrolment for each school were provided by the provincial Ministry of Education (British Columbia Ministry of Education, n.d.). Poisson regression was used to investigate the association between incidence of COVID-19 among students and community incidence.

Univariable and multivariable mixed effects logistic regressions with the school as the random intercept term were performed to identify factors independently associated with in-school acquisition of COVID-19. This multilevel approach was chosen to account for clustering of students within schools. Intra-class correlation (ICC) was calculated to estimate the variation between schools as a proportion of the total variance. The model also controlled for community incidence at the CHSA level in the 2 weeks prior to the case’s symptom onset or, if asymptomatic, testing date. Variables with a *p*-value under 0.10 in univariate analysis (gender, role, school type, masking mandate) and variables identified as relevant in the literature (detection of a variant of concern) were entered into the multivariable model using a stepwise backward approach. Variables that were not statistically significant, did not improve the fit of the model and did not modify the estimates for other variables were removed from the final model. Interaction effects among covariates were tested and added to the model if they were significant. The associations are presented as odds ratios (OR) with 95% confidence intervals (CI). All model assumptions were met and residuals were tested with no significant concerns. In regressions, model assumptions were tested using standard tests and nested models were compared using Akaike information criterion (AIC). Statistical analyses were performed using R version 4.0.3.

### Ethics approval

This analysis was conducted as part of routine public health surveillance activities and was therefore exempt from ethics review and approval.

## Results

**Fig. 1:**
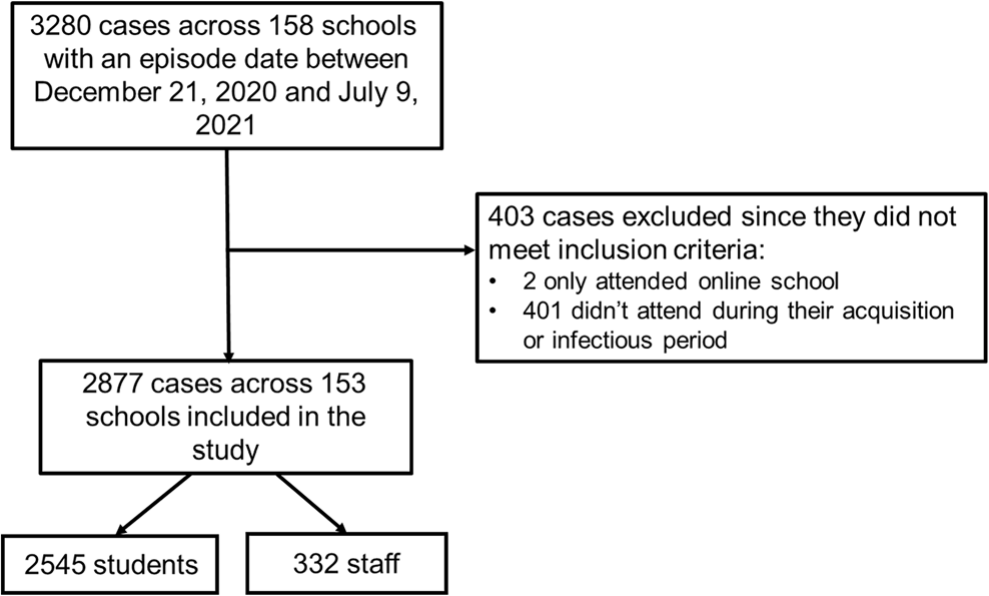
Flow chart for inclusion in the study

There were 3280 cases of COVID-19 among students and staff who attended a K-12 school within the geographic boundaries of Surrey school district during the second half of the 2020–2021 school year. This represents approximately 3.2% of the estimated school population in the study area (*n*~101,900). Of those 3280 cases, 87.7% (*n*=2877) met the inclusion criteria and were included in the review (Fig. 1).

Characteristics of cases included in the study are shown in Table 1. A majority of cases were among students, with 2545 (88.5%) cases being students and 332 (11.5%) cases being staff members. Staff members were predominantly female (*n*=266, 80.1%), and approximately half the students were male (*n*=1351, 53.1%). The mean age was 11.7 (SD: 3.7) and 41.9 (SD: 11) among students and staff, respectively. The proportion of asymptomatic cases at time of assessment was 22.8% (*n*=554) among students and 4.2% (*n*=14) among staff. Overall, 0.6% (*n*=17) cases were hospitalized, including eight students (age range: 5–11 years old). Four adult cases required intensive care, and no deaths occurred.

**Table 1:** Characteristics of students and staff included in the review

	Students, *n* (%)	Staff, *n* (%)	Total, *n* (%)
Number of cases	2545	332	2877
Sex			
Female	1194 (46.9)	266 (80.1)	1461 (50.8)
Male	1351 (53.1)	66 (19.9)	1418 (49.2)
Mean age (standard deviation)	11.7 (3.7)	41.9 (11.0)	15.2 (10.9)
Age category			
5–11	1207 (47.4)	0 (0)	1207 (42.0)
12–18	1335 (52.5)	0 (0)	1335 (46.4)
19 and older	3 (0.1)	332 (100)	335 (11.6)
Asymptomatic at assessment			
No	1991 (78.2)	318 (95.8)	2309 (80.3)
Yes	554 (21.8)	14 (4.2)	568 (19.7)
Outcome			
Hospitalized	8 (0.3)	9 (2.7)	17 (0.6)
ICU admission	0 (0)	4 (1.2)	4 (0.1)
Died	0 (0)	0 (0)	0 (0)

### Association between community incidence and incidence among school students

**Fig. 2:**
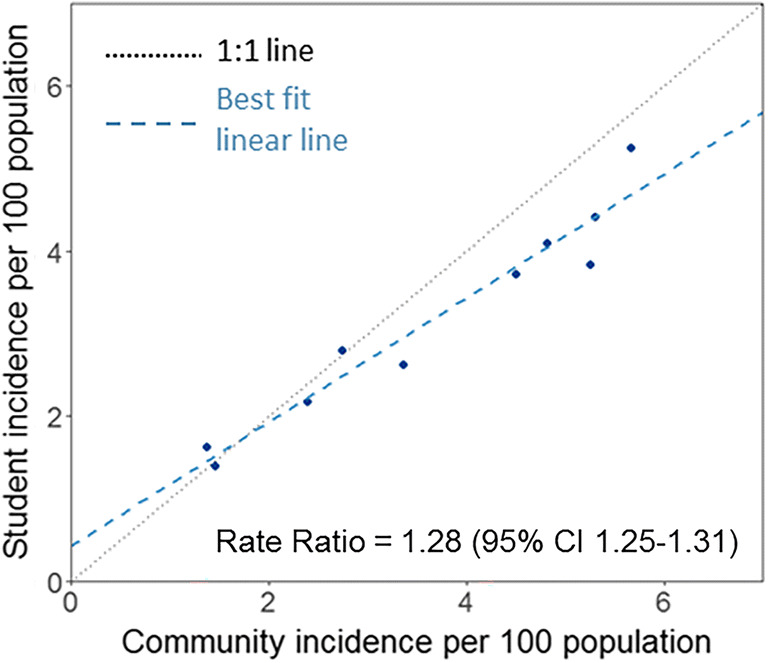
Association between community incidence and incidence in the school setting using a Poisson regression model

Our results show a strong association between COVID-19 incidence in communities surrounding the school and the incidence among school-based individuals, with the risk of student cases increasing by 28% (95% CI: 25–31) for every 1 case per 100 population increase in community incidence (*p*<0.0001) (Fig. 2).

### Transmission in the school setting

Of the 2877 cases included in the study, 9.1% (*n*=262) had evidence of in-school acquisition, and 74.5% (*n*=2142) had evidence of out-of-school acquisition. The source of acquisition could not be assessed based on epidemiological and laboratory data for 16.4% (*n*=473) of cases.

Compared to students, staff were more likely to have acquired in the school setting (13.0% vs 8.6%, *p*=0.015) (Table 2). Staff were also more likely to have introduced COVID-19 in schools; in clusters where the primary case could be identified (*n*=95), 72.6% had a student and 27.4% had a staff member as the primary case (Table 3), despite staff members representing only 11.5% of all cases in the school setting. Of the 266 in-school transmission events identified, the majority occurred either from student to student (*n*=122, 45.9%) or from staff to student (*n*=38, 14.3%) (Table 4).

**Table 2:** Source of acquisition of COVID-19 for cases who attended or worked at a K-12 school during the study period

	Student cases		Staff cases		Total		*P*-value^1^
	*n*	%	*n*	%	*n*	%	
Source of acquisition							<0.001
School	219	8.6	43	13.0	262	9.1	
Out-of-school	1,956	76.9	186	56.0	2,142	74.5	
Undetermined	370	14.5	103	31.0	473	16.4	

^1^Pearson’s chi-squared (*χ*^2^) test

**Table 3:** Characteristics of clusters with evidence of transmission in the school setting

	Number of clusters	
	*n*	%
Number of clusters	126	-
Size of clusters		
Median (interquartile range)	2 (1)	
Mean (standard deviation)	3 (5.3)	
Min, max	2–61	
Number of cases per cluster		
2 cases	91	72.2
3–5	28	22.2
6+	7	5.6
Role of primary case		
Student (< 12 y/o)	34	27.0
Student (≥ 12 y/o)	35	27.8
Staff	26	20.6
Unknown primary case	31	24.6
Variant of concern		
Alpha	49	38.8
Delta	4	3.2
Gamma	16	12.7
VOC not detected	57	45.2
School level		
Elementary	71	56.3
Secondary	45	35.7
Both elementary and secondary	10	7.9
School size		
<500 students	51	40.5
500–999 students	23	18.3
1000+ students	52	41.3
School type		
Public	101	80.2
Independent	25	19.8

**Table 4:** Directionality of transmission events in the school setting

	Transmission events	
	*n*	%
Direction of transmission		
Student to student	122	44.2
Student to staff	25	9.1
Staff to staff	14	5.1
Staff to student	38	13.8
Could not be assessed	77	27.9

We identified 126 clusters where in-school transmission occurred during the second half of the 2020–2021 school year (Table 3). The size of clusters was small, with a median size of two cases (range 2–61), and 72.2% (*n*=91) of clusters leading to only one secondary case in the school setting. Of the 126 clusters identified, 71 (56.3%) were in elementary schools, 45 (35.7%) were in secondary schools, and 10 (7.9%) were in combined elementary and secondary schools. Additionally, 101 (80.2%) clusters occurred in public schools, and 25 (19.8%) occurred in independent schools.

During the study period, only one cluster met the criteria for outbreak declaration, which prompted a mass testing campaign and resulted in the school being ordered to close for 14 days. In this cluster, 61 cases had either transmitted or acquired SARS-CoV-2 in school, including 21 detected through mass testing. Overall, 15 different classrooms from kindergarten to Grade 7 were affected.

### Risk factors for in-school acquisition of COVID-19

The explanatory variables included in the regression analysis to identify risk factors for in-school acquisition are summarized in Table 5. The ICC shows that 29.3% of the variability in the risk of in-school acquisition was attributable to school-level variation (ICC=0.293). The final model suggests three significant risk factors for in-school acquisition of COVID-19: being male (aOR: 1.59; 95% CI 1.17–2.17), being a staff member (aOR: 2.62, 95% CI 1.64–4.21) and attending or working in an independent school (aOR: 2.28; 95% CI 1.13–4.62).

**Table 5:** Univariable and multivariable analysis of the risk factors for in-school acquisition of COVID-19 with school as a random effect^1^

Case and school characteristics	Univariable analysis		Multivariable analysis	
	Odds ratio	95% CI	Adjusted odds ratio	95% CI
Gender				
Female (ref. group)	*-*	*-*	*-*	*-*
Male	1.37	1.02–1.83	**1.59**	1.17–2.17
Role				
Student (< 12 y/o) (ref. group)	*-*	*-*	*-*	*-*
Student (≥ 12 y/o)	0.97	0.66–1.44	0.99	0.67–1.47
Staff	**2.42**	1.54–3.80	**2.62**	1.64–4.21
School size				
<500 students (ref. group)	-	-	-	-
500–999 students	0.90	0.45–1.78	-	-
1000+ students	1.31	0.62–2.74	-	-
Variant of concern detected				
No (ref. group)	-	-	*-*	*-*
Yes	0.91	0.67–1.23	*-*	*-*
Vaccination status^2^				
Unvaccinated (ref. group)	-	-	*-*	*-*
Partially vaccinated	**3.46**	1.44–8.31	-	-
School type				
Public (ref. group)	-	-	*-*	*-*
Independent	**2.24**	1.11–4.50	**2.28**	1.13–4.62
Mask mandate				
Pre mask mandate	**1.59**	1.16–2.17	**-**	-
Post mask mandate (ref. group)	-	-	-	-

^1^Multivariable regressions are adjusted for community incidence in the two weeks prior to the case’s symptom onset or, if asymptomatic, testing date

^2^Vaccination status was removed from the multivariable model since all vaccinated cases were staff members

## Discussion

We found that K-12 schools represented a low risk for transmission of COVID-19 at the time of study, with 9.1% of cases identified in the second half of the 2020–2021 school year having evidence of in-school acquisition. This is consistent with the results from studies in other countries, including the USA, England, Ireland and Germany, which have found that transmission of SARS-CoV-2 in educational settings is uncommon (Ismail et al., 2021; Ladhani et al., 2021; Theuring et al., 2021; White et al., 2021; Zimmerman et al., 2021).

Most transmission events took place from student to student, which is expected considering that students represent a majority of the school population. Nonetheless, staff members were identified as the primary case for a fifth of clusters, despite representing only 11.5% of school cases during the study period. While this might suggest that transmissibility increases with age, there is no consensus about this in the published literature (Coffin & Rubin, 2021; Lyngse et al., 2021; Stein-Zamir et al., 2020). Another hypothesis is that behavioural factors might explain the disproportion, as staff members might experience difficulties maintaining physical distancing from children while exercising their functions. They might also come into contact with a higher proportion of the school population compared to students, who are less likely to be in proximity to individuals outside of their learning group.

Compared to their female peers, male individuals in the study were more likely to have acquired in the school setting. This is in line with research among adults, which has suggested that women tend to demonstrate a higher level of compliance with safety measures (de la Vega et al., 2020), and that men tend to engage in more behaviours putting them at risk of contracting COVID-19 (Griffith, 2020). Further work is needed to assess whether similar factors might explain the higher risk of in-school acquisition among male children.

Overall, 35.7% of the clusters were identified in secondary schools, despite those representing only 20.6% of the schools in the study area. This is consistent with findings from other studies suggesting higher transmission rates of SARS-CoV-2 in middle and high schools, potentially due to adolescents being more likely than children to interact with non-household members outside school (CDC, 2020; Larosa et al., 2020).

Our results also suggest a higher likelihood of in-school acquisition of COVID-19 for individuals who worked at or attended an independent school. This category includes a wide range of schools, with the common characteristic of not being operated by the government. Findings from internal outbreak investigations identified different factors that may have led to the higher rate of transmission observed in this setting; those included a reliance on carpooling between different households to get to school, and multiple members from the same household attending the same school while infectious. Nonetheless, it might not be possible to identify specific factors that apply to all independent schools, since these schools present a wide diversity in their mode of operation and the communities they serve.

Only one cluster of more than 10 cases was detected during the study period. This suggests that while in-school acquisition of COVID-19 is uncommon, widespread transmission in this setting can occur. This is consistent with literature from other countries, with various outbreak reports in the school setting having been published since the emergence of COVID-19 (Kampe et al., 2020; Lam-Hine et al., 2021; Stein-Zamir et al., 2020).

The second half of the 2020–2021 school year corresponded to a period of high community transmission of COVID-19. The average daily case rate reached 48 cases per 100,000 population in April 2021 in the local health area (LHA) of Surrey, which covers the geographic area where most schools included in the study are located (BCCDC, n.d.-b). This high incidence rate was reflected in schools, with the risk of student cases increasing by 28% for every one case per 100 population increase in community incidence. This confirms findings from similar studies in other countries, which have shown that an increase in community transmission is linked to an increase in the numbers of introductions into schools (Ismail et al., 2021; Russell et al., 2020).

A strength of this study was our ability to leverage an existing public health surveillance infrastructure in order to review epidemiological data on a large number of staff and students over an extended period. Our study also had limitations. Given the rapid developments of the COVID-19 pandemic, our findings are tied to a specific context and might not be generalizable to later stages of the pandemic, including the emergence of new variants. Mass testing was only conducted when widespread transmission had already been identified at a school; therefore, it is likely that the number of reported cases underestimates the true prevalence in school settings. This is especially true for children, who are more likely than adults to be asymptomatic or have a mild course of disease (Sah et al., 2021). This study also does not account for compliance with provincial K-12 measures and characteristics of the schools that may have impacted transmission, such as classroom size and configuration, nature of instructional activity, and ventilation. Moreover, WGS was only available for approximately two thirds of cases.

## Conclusion

Our in-depth epidemiological investigation of all cases reported during the second half of the 2020–2021 school year in BC’s largest school district contributes to the emerging evidence suggesting that in-school transmission of SARS-CoV-2 was uncommon during that time period. Nonetheless, the continuous surveillance of school settings by public health authorities is warranted, especially in the context of emerging variants with increased transmissibility.

## Contributions to knowledge

What does this study add to existing knowledge?

- Between January and June, 2021, 9.1% of cases of COVID-19 among students and staff who attended a K-12 school within the geographic boundaries of Surrey school district had evidence of in-school acquisition.
- Prevalence of COVID-19 in K-12 school settings is strongly tied to community incidence, with the risk of student cases increasing by 28% (95% CI: 25–31) for every 1 case per 100 population increase in community incidence.
- Being male, being a staff member, and attending or working in an independent school are associated with in-school acquisition of COVID-19.

What are the key implications for public health interventions, practice or policy?

- Our study provides support for schools to remain open for in-person instruction with appropriate mitigation measures in place during the COVID-19 pandemic.
- Mitigation measures that focus on reducing the risk of in-school acquisition could benefit from targeting male individuals and staff members. Independent schools might also benefit from additional public health support to reduce the risk of in-school transmission.
- Further research is needed to better understand how the risk differs according to context, such as the circulation of different variants of the virus, changing vaccination rates, and mitigation measures in place in the school setting.

## Appendix 1. Summary of provincial COVID-19 health and safety guidelines for K-12 settings

- Public health measures
  - ○ Includes orders from the Provincial Health Officer, improved testing, and contact tracing.
    - During the study period, provincial restrictions on gatherings and events, in addition to sector-specific guidance and orders, were in place during a declared state of emergency (archived here: https://www2.gov.bc.ca/gov/content/health/about-bc-s-health-care-system/office-of-the-provincial-health-officer/current-health-topics/covid-19-novel-coronavirus#archived-orders).
      - In response to meeting specific vaccination targets, case counts and hospitalization rates, the province moved to Step 1 of the provincial restart plan (https://www2.gov.bc.ca/gov/content/covid-19/info) on May 25, 2021, that loosened restrictions on gatherings, travel, businesses, offices and workplaces, sports and exercise. These were further loosened on June 15, 2021, when the province moved to Step 2 of its restart plan.
      - Educational events/activities were exempt from events and gatherings orders and instead followed other measures such as physical distancing as referenced in the K-12 guidance. Staff gatherings were subject to safety measures outlined in WorkSafeBC, generally preferring virtual gatherings over in-person gatherings and where in-person gatherings were in place, following safety measures such as maximizing physical distancing.
    - Testing eligibility included those who had symptoms consistent with COVID-19. Asymptomatic close contacts in the Fraser Health region were recommended to get tested. Sample collection was done using a nasopharyngeal swab or saline gargle.
    - Public health engaged in contact tracing of school-associated cases.
- Environmental measures
  - ○ Includes being outdoors, physical barriers, visual cues for traffic flow and more frequent cleaning and disinfection.
  - Ventilation systems should be checked to ensure working properly, open windows as weather permits.
  - General cleaning and disinfecting at least once every 24 h, high-touched surfaces at least twice every 24 h.
  - Use floor markings and posters to address traffic flow throughout the school.
  - Barriers can be installed in places where physical distancing cannot regularly be practised and a person is interacting with numerous individuals outside of a cohort.
- Administrative measures
  - ○ Includes changes in scheduling and work practices, health and wellness policies, and placing students and staff in cohorts.
  - Cohorts (groups of students and staff who remain together throughout a school term) were in place to minimize the number of individual interactions. Elementary and middle school cohorts could be up to 60 people, secondary schools up to 120 people and could be composed of both students and staff.
  - Physical distancing was to be practised by ensuring 1–2 m of space between people from different cohorts, and avoiding prolonged close contact within cohort.
  - Interactions between cohorts were permitted with safety measures in place to maximize use of physical distancing and minimize the number of interactions between cohorts (ex. staggered recess or lunch breaks); however, school gatherings were expected to be limited to within-cohort gatherings and be infrequently in person, pursuing alternatives such as virtual gatherings.
  - Extracurricular activities could occur with physical distancing measures in place. Inter-school events, overnight or international field trips were not recommended.2
  - School bus transportation was permitted with safety measures (ex. cleaning, disinfection) in place.2
  - Food services were permitted for learning and for food service provision. Homemade food items were not recommended to be made available for other students (ex. bake sales).2
  - Visitors were allowed, however they were recommended to be limited to those supporting activities that are of benefit to student learning and well-being.
- Personal measures
  - ○ Includes staying home when sick, and practising physical distancing, hand hygiene and respiratory etiquette.
  - Cases, close contacts and those who travelled outside of Canada within the last 14 days were to stay home.2
  - Daily health checks were recommended with individuals developing new symptoms advised to stay home with return to school dependent on factors such as COVID-19 test results (if recommended), resolution of symptoms.2
  - Hand hygiene and respiratory etiquette were recommended.
- Personal protective equipment
  - ○ Includes gloves and masks.
  - For the whole study period:
    - Non-medical masks were indicated for middle and secondary students and staff when physical distancing cannot be consistently practised, and a person is interacting with people outside of their cohort.2
    - Masks were provided by the schools as needed.2
    - Masks were not required when outdoors or when the person is eating or drinking.2
    - Gloves were intended for practices such as clean-up of bodily fluids, but not expected to be part of the regular course of work or study.
  - Since February 4, 2021:
    - K-12 staff and middle/secondary students should wear a mask indoors at school except when:
      - Sitting or standing at their seat or workstation in a classroom or learning space,
      - There is a barrier in place,
      - Eating or drinking.
    - K-12 staff and middle/secondary students should wear a mask on buses.
  - Since March 27 (Surrey schools) and March 30 (provincial), 2021
    - All staff, adult volunteers and visitors, and all Grade 4 to 12 students should wear a non-medical mask or face covering at all times while indoors at school, subject to exceptions such as if the person is eating or drinking.

## Appendix 2. Criteria to assess evidence of in-school acquisition of COVID-19

Cases were classified as having evidence of in-school acquisition if they:

1. Attended school during their acquisition period,
2. Did not have any other known possible acquisition source (e.g. household or community), and
3. Were in close contact with an infectious case of COVID-19 in the school setting, or their acquisition period overlapped with the infectious period of another case in the same classroom or administrative area

Other possible situations:

**Table:**

Situation	Decision
Multiple cases in the same classroom or administrative area have a symptom onset within two calendar days and do not have any known exposure outside of the school setting.	The cases are classified as having evidence of in-school acquisition owing to suspicion of a ‘silent’ primary case.
Cases have been exposed both in school and in another setting and the directionality of transmission cannot be assessed due to one or more cases being asymptomatic.	A Medical Health Officer is consulted to determine whether the case should be classified as having evidence of in-school acquisition based on the following hierarchy of exposure likelihood: household contacts were categorized as the most likely source of infection, followed by any known exposure in community settings (e.g. overnight camping trip where other individuals acquired COVID-19) and last, school settings. Other factors, such as the identification of an exposure source for the case’s contacts, are also taken into account to determine direction of transmission.
School and non-school acquisition sources are found to be equally likely.	The case is labelled as having evidence of in-school acquisition.
